# Inhibiting the uPAR/FPR1 interactions reduces blood-retinal barrier breakdown and improves retinal function in a rat model of diabetes

**DOI:** 10.3389/fnins.2026.1872366

**Published:** 2026-07-01

**Authors:** Alessio Canovai, Rosario Amato, Alberto Melecchi, Maria De Fenza, Vincenzo Pavone, Daniele D’Alonzo, Maurizio Cammalleri, Massimo Dal Monte

**Affiliations:** 1 Department of Biology, University of Pisa, Pisa, Italy; 2 Department of Chemical Sciences, University of Naples Federico II, Naples, Italy; 3 Interdepartmental Center Pisa Neuroscience “PiNeuro”, University of Pisa, Pisa, Italy

**Keywords:** apoptosis, electroretinogram, neuroinflammation, neuroprotection, retinal gliosis, streptozotocin

## Abstract

Diabetic retinopathy (DR) is a leading cause of blindness characterized by early neurovascular damage driven by hyperglycemia-induced mechanisms, including inflammation. The system composed of the urokinase-type plasminogen activator (uPA) and its receptor (uPAR) has previously emerged as a potential regulator of the pro-inflammatory events in DR, possibly through the interaction of uPAR with its lateral partners, such as formyl peptide receptors (FPRs). This study explored whether the inhibition of uPAR/FPR1 crosstalk may reduce early neurovascular alterations in DR by targeting inflammation. To this aim, the new FPR1 antagonist N-19004 was tested in a rat model of streptozotocin-induced diabetes. N-19004 was administered subcutaneously for 7 days at 1 month from diabetes onset. Immunofluorescence, RT-qPCR, Western blot and Evans blue perfusion were performed to evaluate the effects of N-19004 on inflammation, reactive gliosis, blood-retinal barrier (BRB) integrity and apoptosis. In addition, electroretinogram (ERG) was used to assess N-19004 efficacy on retinal function. N-19004 inhibited the activation of inflammation-related transcription factors, including nuclear factor kappa-light-chain-enhancer of activated B cells and signal transducer and activator of transcription 3, leading to reduced interleukin-1β and tumor necrosis factor-*α* expression. The attenuation of inflammatory processes resulted in reduced glial activation, as indicated by lower glial fibrillary acidic protein expression and Müller cell gliosis. The anti-inflammatory activity of N-19004 was accompanied by decreased BRB breakdown, as demonstrated by N-19004-mediated reduction of vascular endothelial growth factor, increased levels of tight junction components and diminished vessel leakage. The amelioration of BRB integrity was associated with reduced activation of caspase 3 and partial preservation of scotopic ERG a- and b-wave amplitudes, thereby improving retinal viability and function in N-19004-treated STZ rats. These results support the possible involvement of uPAR/FPR1 interactions in the regulation of DR-related inflammation and suggest a novel therapeutic target for the management of the early phases of disease.

## 1 Introduction

Diabetic retinopathy (DR) is a leading cause of blindness that occurs as a common complication of the diabetic condition. The progressive vision loss occurring in DR arises from a complex interplay of hyperglycemia-induced mechanisms, driving both neuronal damage and vascular alterations that disrupt retinal function from the earliest stages of the disease (Sinclair and Schwartz, 2019). Among these mechanisms, multiple biochemical pathways increase the production of reactive oxygen species (ROS) and pro-inflammatory cytokines, that in turn contribute to the onset of oxidative stress and inflammation, causing neuronal injury and disrupting the integrity of the blood-retinal barrier (BRB; Al-Kharashi, 2018). In turn, BRB breakdown increases vascular permeability, allowing toxic proteins and inflammatory cells to leak into the retinal tissue, thereby exacerbating neuronal dysfunction and triggering retinal hypoxia, which accelerates the disease progression toward its later proliferative stages (Zhou and Chen, 2023). In this respect, experimental evidence suggests that preserving BRB integrity under hyperglycemic conditions may represent a viable strategy to limit early retinal damage, highlighting the potential value of therapeutic interventions targeting the early stages of DR (Giurdanella et al., 2017). Although current pharmacological therapies are effective to a certain extent, they require prolonged treatment regimens and invasive administration routes. Moreover, the currently available treatments for DR primarily target advanced proliferative stages where retinal dysfunction is profoundly compromised (Seo et al., 2025). Therefore, there is an urgent need to investigate the mechanisms driving the early stages of DR to identify alternative therapeutic strategies that possibly intervene in early phases to prevent or retard DR progression towards more severe and irreversible stages.

Among the pathways involved in the progression of DR, the system composed of the urokinase-type plasminogen activator (uPA) and its receptor (uPAR) has previously emerged as a particularly attractive target for its major involvement as a mediator of hyperglycemia-related vascular alterations and BRB breakdown (El-Remessy et al., 2003; Navaratna et al., 2008; El-Remessy et al., 2013; Cammalleri et al., 2017). In this system, uPA interacts with uPAR and catalyzes the conversion of plasminogen into its active form plasmin, promoting extracellular matrix degradation during pro-angiogenic phenomena (Leth and Ploug, 2021). The involvement of the uPA/uPAR system in mediating the microvascular disorders occurring in early DR is supported by its upregulation under hyperglycemic conditions, where the activation of uPA/uPAR may generate a proteolytic cascade that negatively impacts BRB integrity and increases vascular permeability (El-Remessy et al., 2003; Navaratna et al., 2008; Yang et al., 2010; Yang et al., 2012; Motta et al., 2016; D'Alonzo et al., 2020).

Besides its pro-angiogenic role, the uPA/uPAR system may regulate proteolysis-independent cellular processes through interactions with lateral partners such as integrins and G protein-coupled receptors, including formyl peptide receptors (FPRs; Cammalleri et al., 2019). These interactions can activate intracellular signaling cascades that promote the expression of pro-inflammatory cytokines and chemokines, thus implicating the uPA/uPAR system as a potential regulator of the inflammatory mechanisms contributing to microvascular damage in early DR. In line with this hypothesis, our previous studies demonstrate that the pharmacological inhibition of uPAR/FPRs crosstalk significantly prevents BRB breakdown and retinal dysfunction in experimental models of DR, likely through anti-inflammatory effects (Cammalleri et al., 2017; Cammalleri et al., 2017).

Given the important role of FPRs as lateral partners of uPAR and the relevance of inhibiting their interactions to prevent the activation of uPAR downstream signaling, we designed a novel uPAR antagonist, called N-19004, that strongly interferes with FPR1-promoted cell migration *in vitro*. The systemic administration of N-19004 in a mouse model of choroidal neovascularization, a model mimicking neovascular age-related macular degeneration (AMD), reduced the size of laser-induced neovascular lesions and improved oedema reabsorption by combining anti-angiogenic and anti-inflammatory activities (De Fenza et al., 2024). In this line, the administration of this compound in the rd10 mouse model of retinitis pigmentosa reduces photoreceptor degeneration possibly through an anti-inflammatory effect, thus further supporting the role of uPAR/FPR1 interaction in regulating the inflammatory processes in the diseased retina (Amato et al., 2025).

Based on these studies, we herein assessed the therapeutic potential of N-19004 in counteracting DR in the rat model of streptozotocin (STZ)-induced diabetes, a well-established model of type I diabetes. We hypothesized that the inhibition of uPAR/FPR1 interaction may be sufficient to mitigate the early neurovascular damage in this model through the inhibition of inflammatory processes. In this study, the anti-inflammatory activity of N-19004 in STZ rats was evaluated by analyzing the expression of key pro-inflammatory factors and markers of gliosis using qPCR. The correlation between N-19004-related anti-inflammatory activity and its effect on early vascular alterations was evaluated by assessing the levels of inter-endothelial tight junction components and BRB permeability. In addition, the protective efficacy of N-19004 on retinal cell viability and function was evaluated by assessing the presence of apoptotic processes and measuring retinal activity by electroretinogram (ERG), respectively.

## 2 Materials and methods

### 2.1 Animals

Animals were used in accordance with the Association for Research in Vision and Ophthalmology statement for the Use of Animals in Ophthalmic and Vision Research. This study meets also the requirements of the European Communities Council Directive (2010/63/UE) and the Italian guidelines for animal care (DL 26/14). The principles of the 3Rs for the ethical use of animals in scientific research were applied to limit both the number and the suffering of the animals. Male Sprague Dawley rats of 8 weeks old and weighting about 200 g were purchased from Envigo Italy (San Pietro al Natisone, Italy) and used for the experiments. Rats were maintained in a regulated environment characterized by 23 ± 1 °C, 50 ± 5% humidity and 12 h light/dark cycles with lights on at 08:00 a.m. Animals were provided with a standard diet and water ad libitum. A total of 40 rats was used for the study. The animals were divided into four groups containing 10 rats each: healthy controls, untreated STZ-injected animals, STZ-injected rats treated with N-19004 at either low or high dose (see below). In all procedures requiring anesthesia, rats were anesthetized with an intraperitoneal injection of 30 mg/kg sodium pentobarbital. In contrast, for procedures requiring animal euthanasia and subsequent tissue collection, animals received a lethal intraperitoneal injection of sodium pentobarbital at 200 mg/kg. Death was confirmed by monitoring animals for clinical signs including cessation of heartbeat, respiratory arrest and no response to toe pinch.

### 2.2 Rat model of streptozotocin-induced diabetes and treatments

After an overnight fasting, rats received an intraperitoneal injection of 65 mg/kg STZ (Sigma-Aldrich, St. Louis, MO, USA) diluted in citrate buffer, pH 4.5. Age-matched rats were injected with vehicle and considered as controls. To confirm diabetic induction 3 days after the injection of STZ, blood glucose was measured by tail sampling using a OneTouch Ultra glucometer (LifeScan Inc., Milpitas, CA, USA). Rats displaying blood glucose levels equal to or higher than 250 mg/dL were considered diabetic. Blood glucose was regularly measured once a week over the entire experimental period. After 30 days from the induction of diabetes, two groups of STZ-injected animals underwent N-19004 treatment for 7 days. N-19004 was obtained through a synthetic strategy initially established as a five-step procedure (De Fenza et al., 2025) and subsequently refined into a more convenient four-step route (De Fenza et al., 2024). N-19004 solutions were prepared at two different concentrations (3 and 6 mg/mL), in order to be administered at either 6 mg/kg (low dose) or 12 mg/kg (high dose) by subcutaneous injections once daily. This range of treatment dosages was chosen based on previous studies showing protective and anti-inflammatory effects of N-19004 in mouse models of retinal degeneration (De Fenza et al., 2024; Amato et al., 2025). The doses were adjusted for interspecies drug dosage translation by normalizing to body surface area, in order to determine the corresponding dosages for rats (Nair and Jacob, 2016). The treatment timing was based on previous studies in the rat STZ model using other uPAR antagonists (Cammalleri et al., 2017; Cammalleri et al., 2017), whose structure was used as a reference for designing N-19004 (De Fenza et al., 2024).

### 2.3 Quantitative real-time PCR

For molecular analyses, four rats per group were euthanized as described above and eyes were enucleated. Retinas were dissected and stored at −80 °C until further processing for either qPCR or Western blotting (see below). Each sample for the molecular analyses included a single retina. Total RNA from four samples per group was extracted using a column-based isolation kit (RNeasy Mini Kit; Qiagen, Valencia, CA, USA), purified and resuspended in RNAse-free water. Total RNA content was quantified by spectrophotometry (BioSpectrometer basic; Eppendorf AG, Hamburg, Germany). First-strand cDNA was synthesized from an input of 1 μg RNA using a commercially available kit (QuantiTect Reverse Transcription Kit; Qiagen). qPCR was performed using the SsoAdvanced Universal SYBR Green Supermix (Bio-Rad Laboratories, Inc., Hercules, CA, USA) on a CFX Connect Real-Time PCR detection system connected to the software CFX manager (Bio-Rad Laboratories). Forward and reverse primer pair sequences were adopted from previous studies (Cammalleri et al., 2017; Jungwirth et al., 2009; Pesce et al., 2021; Yoshimoto et al., 2024; see Table 1 for the list of assessed genes and related primers). Target genes were run concurrently with ribosomal protein L13a (Rpl13a) used as a housekeeping gene. The stability of the housekeeping gene across the experimental conditions was checked using the method developed by Mane and colleagues (Mane et al., 2008). This analysis demonstrated that the Ct values of Rpl13a were normally distributed in each group and displayed comparable mean values between groups with a very low standard deviation (control: 19.88 ± 0.23, STZ: 19.83 ± 0.17, + low: 19.87 ± 0.20, + high: 19.96 ± 0.15), thus acknowledging Rpl13a as a reliable housekeeping gene. Gene expression was calculated using the relative threshold cycle (ΔΔCt) method and expressed as fold increase.

**Table 1:** List of primer sequences used for RT-qPCR.

Gene	Forward primer (5′ → 3′)	Reverse primer (5′ → 3′)
uPAR	TTGGATGTTCCTACGAAGAGACG	GTAACTCCGGTTTCCCAGCA
FPR1	GTTTCCGCATGAAACGCACT	CATGACCAGGCTGACGATGT
FPR2	GCTTCACAATGCCCATGTCC	ACTCGTAAGGGACGACTGGA
FPR3	TCCCTTTCAACTGGTTGCCC	GCCAATGAGTTGGTTGGCATA
IL-1β	GTGATGTTCCCATTAGACAGC	CTTTCATCACACAGGACAGG
TNF-α	CTCAAAACTCGAGTGACAAGC	CCGTGATGTCTAAGTACTTGG
GFAP	TGACGCCTCCACTCCGTGCC	CATCTCCGCACGCTCGCTGG
VEGF	CAATGATGAAGCCCTGGAGTG	AGGTTTGATCCGCATGATCTG
Claudin-1	GTTTCATCCTGGCTTCGCTG	CTTTGCGAAACGCAGGACAT
Claudin-5	TACTCAGCACCAAGGCGAAC	TTCCCACATCGGTCTTTCCG
ZO-1	AGTCTCGGAAAAGTGCCAGG	GGGCACGATACCAACCATCA
Caspase 3	AATTCAAGGGACGGGTCATG	GCTTGTGCGCGTACAGTTTG
Rpl13a	GGATCCCTCCACCCTATGACA	CTGGTACTTCCACCCGACCTC

FPR, Formyl peptide receptor. GFAP, Glial fibrillary acidic protein. IL, Interleukin. Rpl13a, Ribosomal protein L13a. TNF, Tumor necrosis factor. uPAR, Urokinase-type plasminogen activator receptor. VEGF, Vascular endothelial growth factor. ZO, Zonula occludens.

### 2.4 Western blotting

To perform Western blotting, four samples per group were homogenized in RIPA lysis buffer (Santa Cruz Biotechnology, Dallas, TX, USA) added with phosphatase and proteinase inhibitor cocktails (Roche Applied Science, Indianapolis, IN, USA). The protein content of the extracts was quantified using the Micro BCA protein assay (Thermo Fisher Scientific, Waltham, MA, USA). Thirty micrograms of protein per sample were separated by SDS-PAGE (4–20%; Bio-Rad Laboratories). Afterwards, gels were transblotted onto nitrocellulose membranes (Bio-Rad Laboratories) that were subsequently blocked with 5% skim milk in Tris-buffered saline containing 0.1% v/v Tween 20 for 1 h at room temperature. Membranes were then incubated overnight at 4 °C with the solutions of primary antibodies indicated in Table 2 diluted in blocking solution. Membranes were then incubated for 2 h at room temperature with appropriate HRP-conjugated secondary goat polyclonal anti-rabbit (Bio-Rad Laboratories; 170–6515) or rabbit polyclonal anti-mouse (Sigma-Aldrich; A9044) antibodies, all diluted at 1:5000 in blocking solution. Blots were developed using Clarity western enhanced chemiluminescence substrate (Bio-Rad Laboratories). Images were acquired using the ChemiDoc XRS + (Bio-Rad Laboratories). The optical density (OD) of the target bands was calculated using Image Lab 6.0 software (Bio-Rad Laboratories) and normalized to the OD of either *β*-actin as loading control or total proteins for phosphorylated targets, as appropriate.

**Table 2:** List of primary antibodies used for Western blotting.

Antibodies	Dilution	Source	Catalog
Rabbit monoclonal anti-pNF-kB p65 (Ser 536)	1:1000	Abcam	ab76302
Rabbit polyclonal anti-NF-kB p65	1:1000	Abcam	ab16502
Mouse monoclonal anti-pSTAT3(Tyr 705)	1:200	Santa Cruz Biotechnology	sc-8059
Rabbit monoclonal anti-STAT3	1:1000	Abcam	ab68153
Rabbit monoclonal anti-cleaved caspase 3	1:500	Cell Signaling Technology	9664S
Mouse monoclonal anti-β-actin	1:2500	Sigma-Aldrich	A2228

NF-kB, Nuclear factor kappa-light-chain-enhancer of activated B cells. STAT3, Signal transducer and activator of transcription 3.

### 2.5 Immunofluorescence

Three rats per group were euthanized as described above. Eyes were enucleated and subsequently immersion-fixed in 4% paraformaldehyde in 0.1 M phosphate-buffered saline (PBS) for 2 h at room temperature. Fixed samples were cryo-protected in 25% sucrose in 0.1 M PBS and then stored at 4 °C. Eyes were embedded in cryo-gel medium and cut into 10 μm-thick cross sections by cryostat sectioning. Retinal sections were mounted onto positively charged slides and subsequently immunostained with rabbit monoclonal anti-GFAP (1:400; Abcam; ab207165) or rabbit monoclonal anti-cleaved caspase 3 (1:400; Cell Signaling Technology, Danvers, MA, United States; 9664S) primary antibodies in 0.1% v/v Triton X-100 in 0.1 M PBS overnight at 4 °C. Thereafter, sections were incubated with donkey polyclonal anti-rabbit conjugated with Alexa Fluor 488 secondary antibody (1:200; Thermo Fisher Scientific; A-21206) in 0.1% v/v Triton X-100 in 0.1 M PBS for 2 h at room temperature. After being rinsed, sections were coverslipped with Fluoroshield mounting medium containing 4′, 6-diamidino-2-phenylindole (DAPI; Abcam) and images were acquired at 20 × magnification using an epifluorescence microscope (Ni-E; Nikon-Europe, Amsterdam, The Netherlands) equipped with a 20x plan apochromat objective and a digital camera (DS-Fi1c; Nikon-Europe). The images of retinal sections were then adjusted for brightness and contrast, trimmed, and included in figure panels using Adobe Photoshop (Adobe Photoshop CS3; Adobe Systems, Mountain View, CA, USA).

### 2.6 Detection of retinal vascular leakage by Evans blue dye

Three rats per group were anesthetized as described above and perfused with 0.5% Evans blue dye (Sigma-Aldrich) in 0.1 M PBS through the left ventricle. After perfusion, eyes were subsequently enucleated and the retinas were dissected and flat mounted onto microscope slides. Images of perfused retinas were acquired using an epifluorescence microscope (Ni-E; Nikon Europe) equipped with a 10x plan apochromat objective and a digital camera (DS-Fi1c camera; Nikon Europe).

### 2.7 Scotopic full-field electroretinogram

Animals were dark adapted overnight and manipulated under dim red light. Rats were anesthetized as described above. To obtain bilateral pupil mydriasis, rats received eye drops of 1% tropicamide on both eyes. A heating pad was used to maintain body temperature at 38 °C and balanced saline solution drops were instilled to avoid the clouding of the ocular media. The recording electrodes consisted of silver/silver chloride wires placed under the lower eyelids. Reference and ground electrodes, made by stainless steel needles, were inserted subcutaneously at the mouse scalp or at the tail root, respectively. The electrodes were linked to a two-channel amplifier and a Ganzfield stimulator (Retimax Advanced, CSO, Firenze, Italy) was used to deliver light flashes. Responses from both eyes were collected simultaneously, amplified at 5000 gain and filtered with a bandpass of 1–100 Hz. Scotopic ERG responses were evoked by flashes at 10 cd·s/m^2^ intensity and ERG waveforms were analyzed using a customized program (Retimax Advanced, CSO). In line with the International Society for Clinical Electrophysiology guidelines, the a-wave amplitude was measured from the pre-stimulus baseline to the negative trough of the a-wave, whereas the b-wave amplitude was calculated from the trough of the a-wave to the positive peak of the b-wave.

### 2.8 Statistical analyses

Data were analyzed using Graph Pad Prism 8.0.2 (GraphPad Software, Inc., San Die-go, CA, USA). Shapiro–Wilk test was performed to assess the normal distribution of the data. One-way or two-way ANOVA test followed by Tukey’s or Bonferroni’s multiple comparison *post-hoc* test, respectively, were used to assess differences among groups as appropriate. Body weight and blood glucose data are expressed as mean ± SD of the indicated n values. The other datasets were plotted as box-and-whisker plots, with boxes representing the 25th to 75th percentiles and whiskers indicating the minimum and maximum values. Differences among groups were considered significant when *p* < 0.05.

## 3 Results

### 3.1 Effects of N-19004 administration on body weight and blood glucose

**Figure 1:**
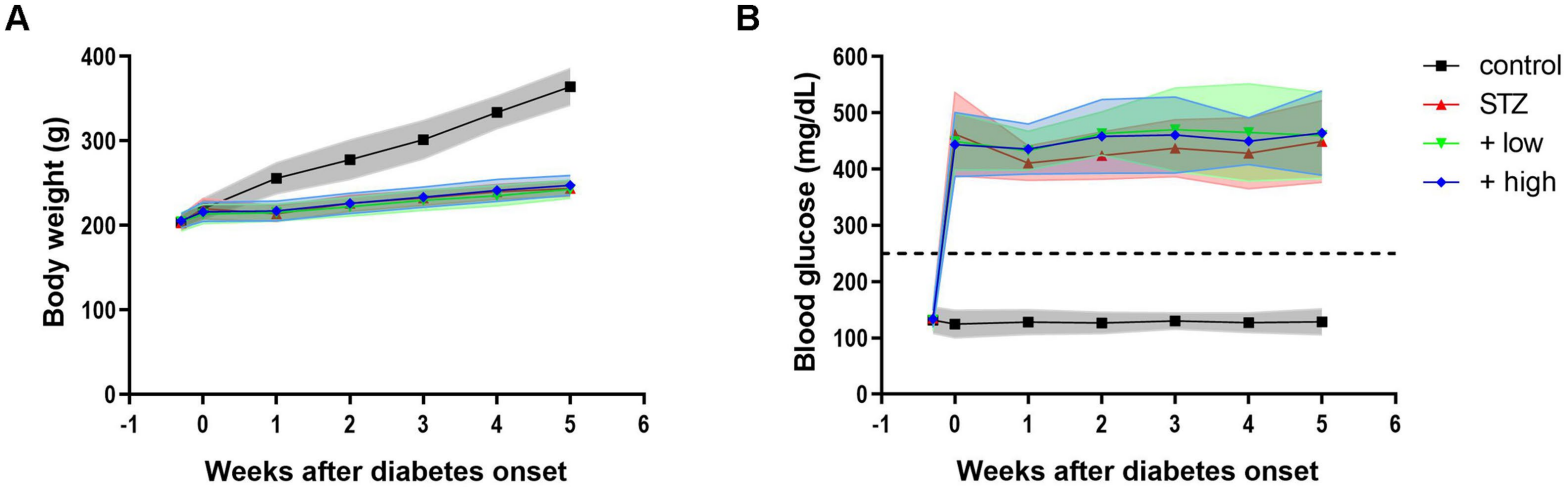
Effects of N-19004 treatment on body weight and blood glucose. Longitudinal evaluation of **(A)** body weight and **(B)** blood glucose levels in control and streptozotocin (STZ) rats either untreated or treated with N-19004 at low or high dose. The dotted line indicates the blood glucose threshold of 250 mg/dL used to establish the diabetic status. Data are expressed as mean with shaded bands indicating SD. Statistical significance was assessed by two-way ANOVA followed by Bonferroni’s multiple comparison *post-hoc* test. *n* = 10 animals per group.

To test whether the treatment with N-19004 affects diabetes-related systemic parameters, body weight and blood glucose levels were measured in control and STZ-injected rats either untreated or treated with N-19004 over the experimental period. At baseline, rats assigned to the control group (203.7 ± 7.13 g) displayed a comparable body weight in relation to the STZ groups, either untreated (203.1 ± 9.07 g, *p* < 0.99) or treated with N-19004 (low dose: 204.5 ± 10.9 g, *p* < 0.99; high dose: 205.4 ± 8.5 g, *p* < 0.97). Control rats displayed a physiological age-dependent gain in body weight over time, which was conversely attenuated in STZ-injected animals with no differences between untreated and N-19004 treated rats (Figure 1A). Following STZ injection, blood glucose levels increased and remained higher than controls up to 5 weeks after diabetes onset. No differences in blood glucose levels were detected between untreated and N-19004 treated STZ rats (Figure 1B).

### 3.2 Effects of N-19004 administration on uPAR/FPRs pathway and its related downstream transcription factors

**Figure 2:**
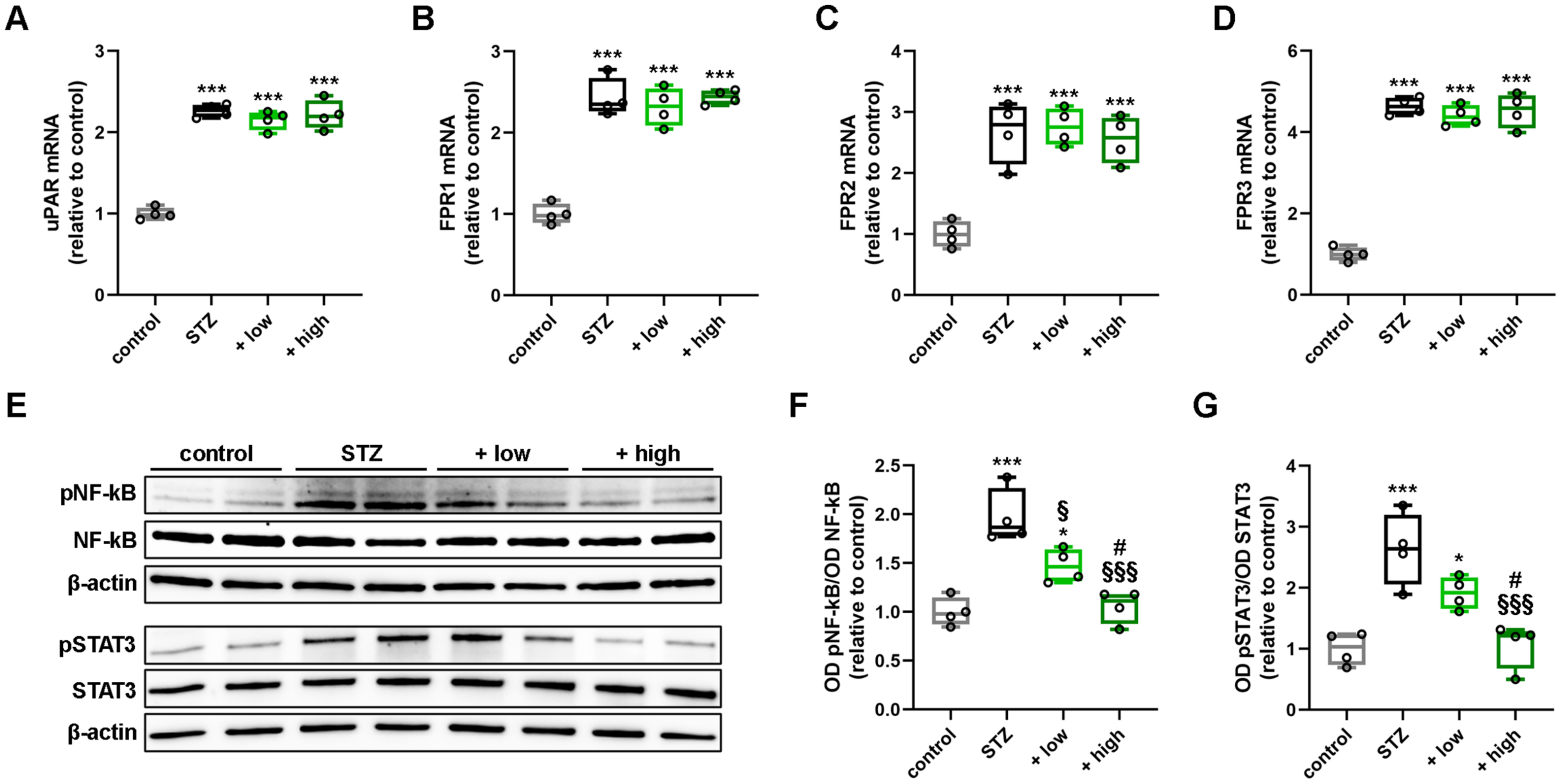
Effects of N-19004 treatment on urokinase-type plasminogen activator receptor (uPAR)/formyl peptide receptors (FPRs) pathway and its related downstream transcription factors. **(A–D)** Quantitative analysis of uPAR **(A)**, FPR1 **(B)**, FPR2 **(C)** and FPR3 **(D)** mRNA levels in retinas from control and STZ rats either untreated or treated with N-19004 at low or high dose. **(E–G)** Representative blots **(E)** and relative densitometric analysis **(F,G)** of phosphorylated nuclear factor kappa-light-chain-enhancer of activated B cells (pNF-kB) at Ser536, NF-kB, phosphorylated signal transducer and activator of transcription 3 (pSTAT3) at Tyr705, and STAT3. The optical density (OD) of pNF-kB and pSTAT3 were normalized to the corresponding OD of their respective unphosphorylated forms (NF-kB and STAT3, respectively). *β*-actin was used as the loading control. Data are expressed as box plots with minimum to maximum whiskers. Differences between groups were assessed using one-way ANOVA followed by Tukey’s multiple comparisons *post-hoc* test. *n* = 4 samples per group. * *p* < 0.05 and *** *p* < 0.001 vs. control; ^§^ *p* < 0.05 and ^§§§^ *p* < 0.001 vs. STZ; ^#^ *p* < 0.05 vs. low dose.

To assess the impact of N-19004 treatment on the uPAR/FPRs pathway, the mRNA levels of uPAR, FPR1, FPR2, and FPR3 were measured by qPCR. A significant increase in uPAR, FPR1, FPR2, and FPR3 mRNA levels was observed in the retinas of untreated STZ rats, as compared to healthy controls. The treatment with N-19004 did not alter the expression of these markers when compared to untreated STZ rats (Figures 2A–D). Despite N-19004 inefficacy in regulating uPAR/FPRs levels, we further evaluated the activation of this pathway after N-19004 treatment by investigating the activation status of some uPAR/FPR1-related downstream transcription factors, including nuclear factor kappa-light-chain-enhancer of activated B cells (NF-kB) and signal transducer and activator of transcription 3 (STAT3). In particular, NF-kB and STAT3 phosphorylation status was evaluated by Western blotting as a readout of their activation. As shown by representative blots and related densitometric analyses in Figures 2E–G, the phosphorylation rate of NF-kB and STAT3 significantly increased in the retinas of untreated STZ rats compared to controls. In contrast, the treatment with N-19044 dose-dependently inhibited the phosphorylation of NF-kB in STZ rats (Figure 2F). On the other hand, the low dose of N-19004 did not significantly affect the levels of pSTAT3 in STZ rats, although a tendency toward a decrease could be detected, whereas the high dose was effective in reducing the phosphorylation rate of STAT3 (Figure 2G).

### 3.3 Effects of N-19004 administration on inflammatory processes

**Figure 3:**
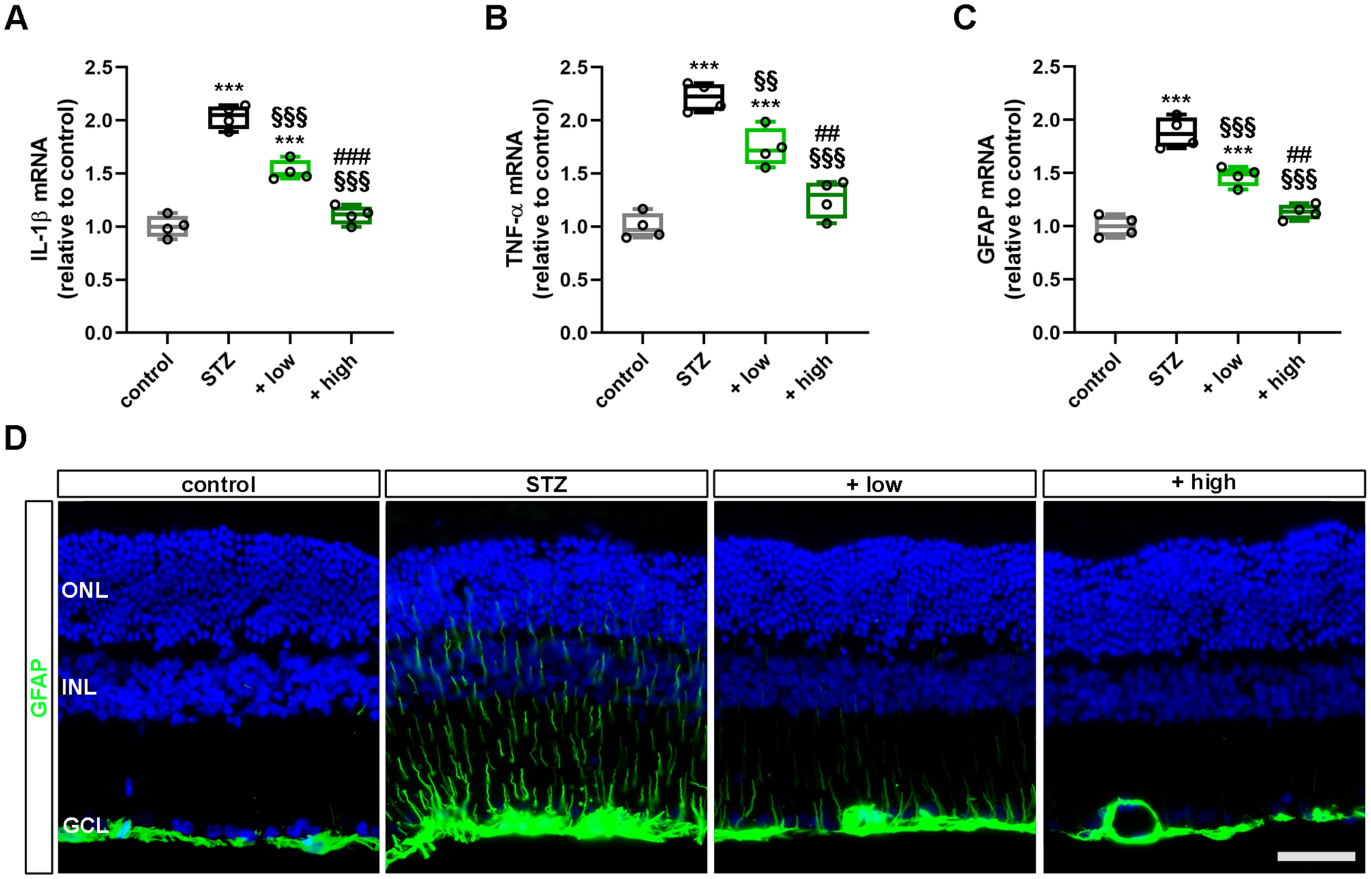
Effects of N-19004 treatment on pro-inflammatory factors and reactive gliosis. **(A–C)** Quantitative analysis of interleukin (IL)-1β **(A)**, tumor necrosis factor (TNF)-*α* **(B)** and glial fibrillary acidic protein (GFAP) **(C)** mRNA levels in retinas from control and STZ rats either untreated or treated with N-19004 at low or high dose. Data are expressed as box plots with minimum to maximum whiskers. Differences between groups were assessed using one-way ANOVA followed by Tukey’s multiple comparisons *post-hoc* test. *n* = 4 samples per group. *** *p* < 0.001 vs. control; ^§§^ *p* < 0.01 and ^§§§^ *p* < 0.001 vs. STZ; ^##^ *p* < 0.01 and ^###^ *p* < 0.001 vs. low dose. **(D)** Representative images of retina sections from control and STZ rats either untreated or treated with N-19004 at low or high dose immunolabeled for GFAP (green). Counterstain with DAPI (blue) was used to visualize retinal cell nuclei. Scale bar: 50 μm. GCL, ganglion cell layer; INL, inner nuclear layer; ONL, outer nuclear layer.

To investigate whether the inhibition of uPAR/FPR1 signaling by N-19004 resulted in an anti-inflammatory effect, qPCR of inflammation-related markers, such as interleukin-1β (IL-1β) and tumor necrosis factor-*α* (TNF-α), was performed. Untreated STZ rats displayed a significant increase in IL-1β and TNF-α mRNA levels compared to their respective controls. Whereas treatment with the low dose of N-19004 resulted in a partial inhibition of IL-1β and TNF-α upregulation, the high dose completely abolished the increase of these markers (Figures 3A,B). As the activation of the inflammatory mechanisms correlates with increased glial reactivity, we further investigated a putative effect of N-19004 on reactive gliosis, using glial fibrillary acidic protein (GFAP) as a well-established marker of reactive glia (Vecino et al., 2016). GFAP mRNA levels increased in the retinas of untreated STZ rats, in respect to healthy controls. This increase was dose-dependently prevented by N-19004 (Figure 3C). The capacity of N-19004 to inhibit reactive gliosis was also confirmed by immunofluorescence analyses of GFAP. As shown in Figure 3D, while GFAP immunostaining in control retinas was limited to the ganglion cell layer (GCL), untreated STZ rats displayed extensive GFAP immunopositive processes spreading across retinal layers, potentially indicating reactive Müller cells. Retinas of STZ rats treated with the low dose of N-19004 showed a prominent reduction of GFAP-positive Müller cell processes and a reduced immunostaining in the GCL. On the other hand, retinas from STZ animals treated with N-19004 at high dose exhibited similar GFAP immunostaining to that of controls, with basal staining in the GCL and barely detectable vertical processes (Figure 3D).

### 3.4 Effects of N-19004 administration on blood-retinal barrier permeability

**Figure 4:**
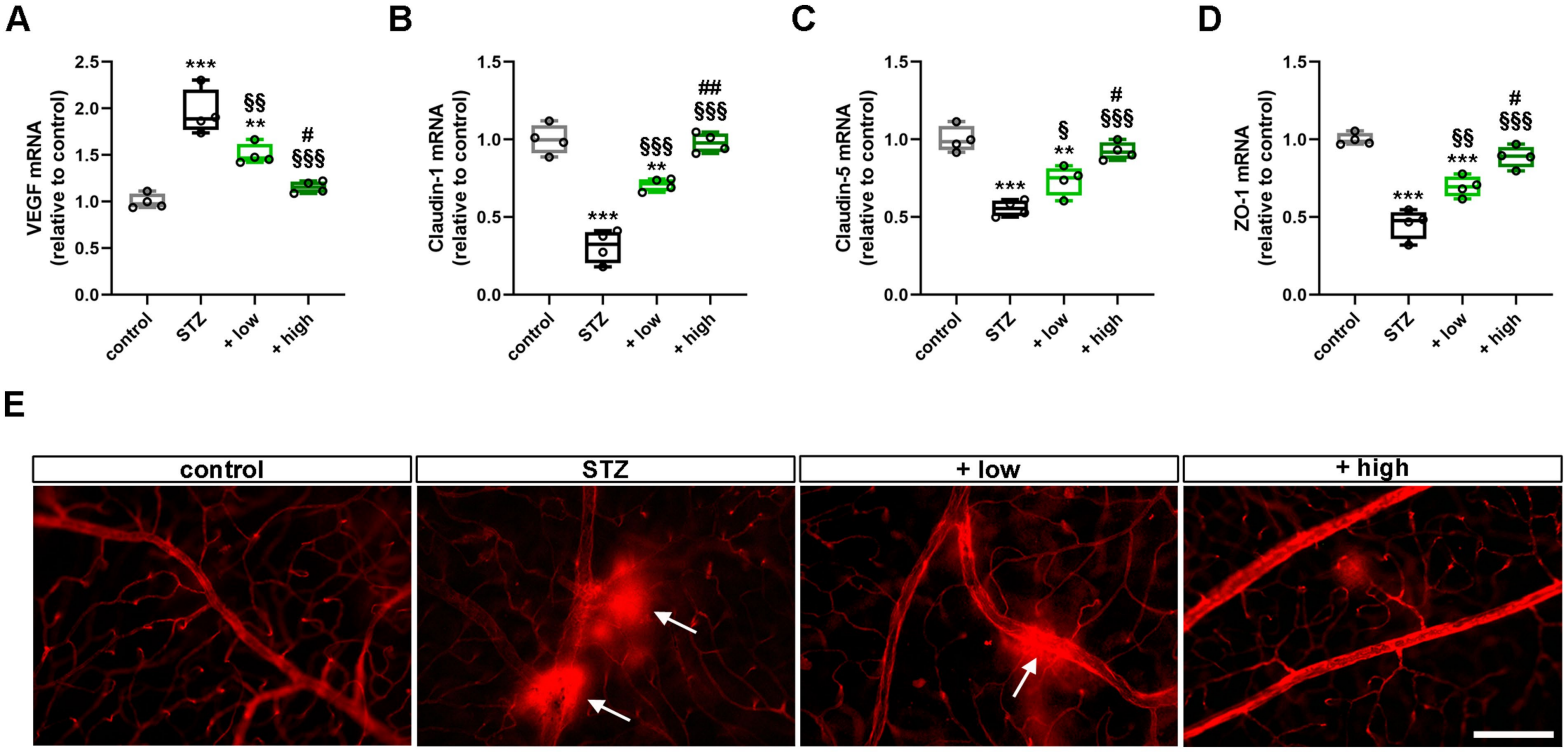
Effects of N-19004 treatment on blood-retinal barrier breakdown. **(A–D)** Quantitative analysis of vascular endothelial growth factor (VEGF) **(A)**, claudin-1 **(B)**, claudin-5 **(C)** and zonula occludens-1 (ZO-1) **(D)** mRNA levels in retinas from control and STZ rats either untreated or treated with N-19004 at low or high dose. Data are expressed as box plots with minimum to maximum whiskers. Differences between groups were assessed using one-way ANOVA followed by Tukey’s multiple comparisons *post-hoc* test. *n* = 4 samples per group. ** *p* < 0.01 and *** *p* < 0.001 vs. control; ^§^ *p* < 0.05, ^§§^ *p* < 0.01 and ^§§§^ *p* < 0.001 vs. STZ; ^#^ *p* < 0.05 and ^##^ *p* < 0.01 vs. low dose. **(E)** Representative images of whole-mounted retinas from control and STZ rats either untreated or treated with N-19004 at low or high dose after Evans blue dye perfusion. White arrows indicate focal areas of blood extravasation. Scale bar: 200 μm.

As reactive Müller cells under hyperglycemia produce some vascular permeability factors, including vascular endothelial growth factor (VEGF), that mediate the early vascular alterations occurring in DR (Rossino et al., 2020), we further assessed whether the anti-inflammatory activity of N-19004 was also accompanied by reduced VEGF expression. In particular, untreated STZ rats displayed increased levels of VEGF mRNA compared to controls (Figure 4A). Notably, the administration of N-19004 inhibited the overexpression of VEGF in STZ rats in a dose-dependent manner. The increased production of VEGF was accompanied by reduced BRB integrity, as evaluated by mRNA levels of inter-endothelial tight junction components, such as claudin-1, claudin-5 and zonula occludens-1 (ZO-1) that, in untreated STZ rats, were significantly diminished as compared with healthy controls (Figures 4B–D). The downregulation of these markers in STZ rats was dose-dependently prevented by the treatment with N-19004. The N-19004-mediated recovery in the levels of tight junction components was also correlated with the reduction of BRB permeability, assessed by Evans blue dye perfusion. Representative images from Evans blue dye-labeled retinas from control or STZ-injected rats either untreated or treated with N-19004 are depicted in Figure 4E. In control rats, Evans blue dye was restricted to the lumen of retinal blood vessels. Conversely, untreated STZ rats displayed clear points of Evans blue dye extravasation as a sign of increased leakage of plasma proteins in the retinal parenchyma. The administration of N-19004 dose-dependently reduced retinal leakage, with the high dose exerting the highest efficacy.

### 3.5 Effects of N-19004 administration on retinal viability and function

**Figure 5:**
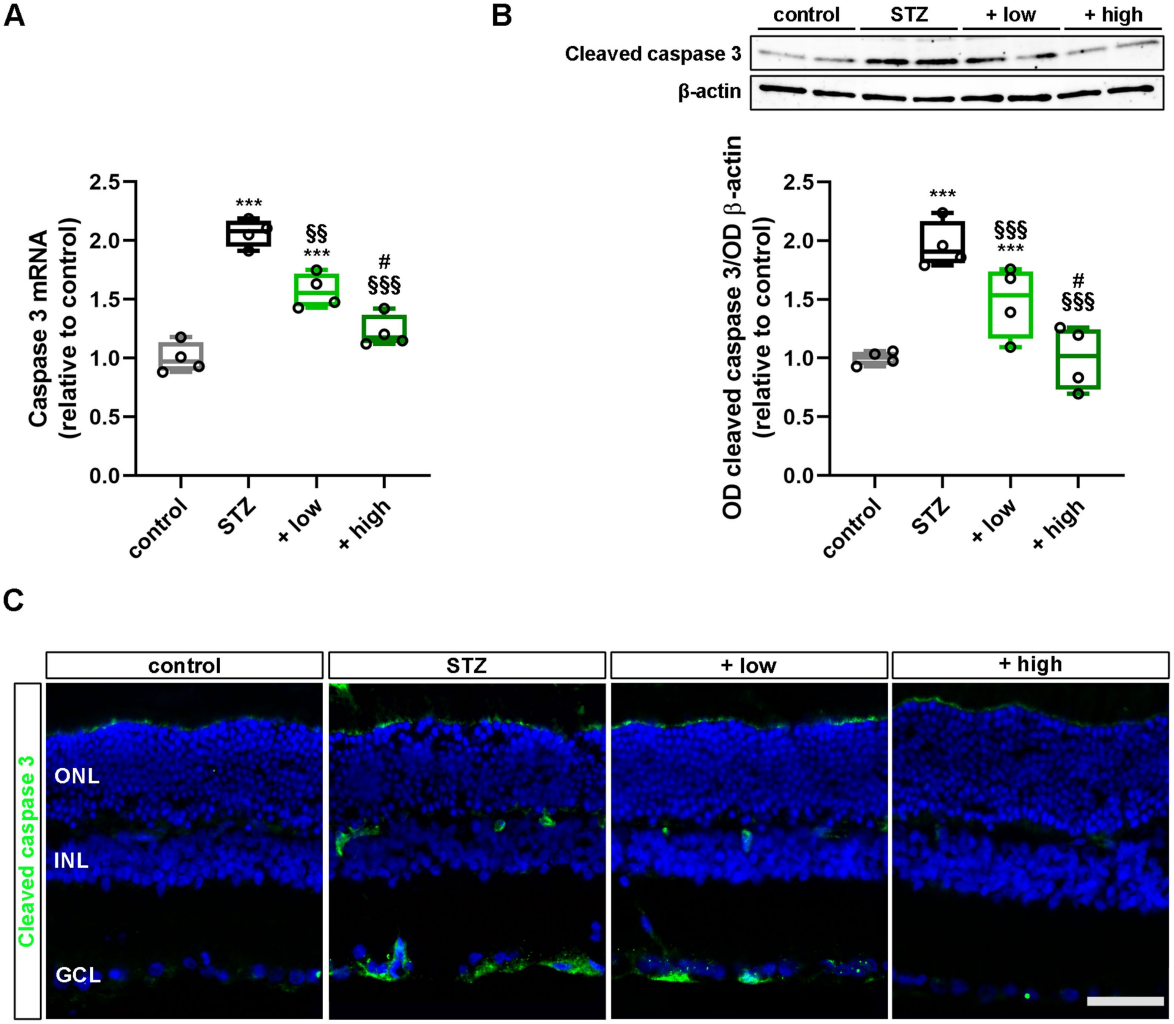
Effects of N-19004 treatment on retinal cell death. **(A)** Quantitative analysis of caspase 3 mRNA levels in retinas from control and STZ rats either untreated or treated with N-19004 at low or high dose. **(B)** Representative blots and relative densitometric analysis of cleaved caspase 3. The OD of the target bands was normalized to the OD of the corresponding β-actin used as loading control. Data are expressed as box plots with minimum to maximum whiskers. Differences between groups were assessed using one-way ANOVA followed by Tukey’s multiple comparisons *post-hoc* test. *n* = 4 samples per group. *** *p* < 0.001 vs. control; ^§§^ *p* < 0.01 and ^§§§^ *p* < 0.001 vs. STZ; ^#^ *p* < 0.05 vs. low dose. **(C)** Representative images of retina sections from control and STZ rats either untreated or treated with N-19004 at low or high dose immunolabeled for cleaved caspase 3 (green). Counterstain with DAPI (blue) was used to visualize retinal cell nuclei. Scale bar: 50 μm. GCL, ganglion cell layer; INL, inner nuclear layer; ONL, outer nuclear layer.

To assess whether N-19004-mediated protection of BRB integrity was also paralleled by reduced retinal cell death, we evaluated the transcriptional levels of caspase 3 and the protein levels of its cleaved form, as indicators of activated apoptotic processes (Feenstra et al., 2013). In particular, untreated STZ rats showed increased caspase 3 mRNA levels, along with a parallel increase in the protein levels of its cleaved form compared to controls (Figures 5A,B). Treatment with N-19004 prevented both the upregulation of caspase 3 mRNA and the increment of its cleaved form in a dose-dependent manner. The reduced activation of caspase 3 was further supported by immunofluorescence analyses, as depicted in Figure 5C. In particular, the retinas of untreated STZ rats displayed several cleaved caspase 3-immunopositive cell profiles, particularly restricted to the GCL and INL, as compared to healthy controls where cleaved caspase 3 immunostaining was nearly absent. The treatment with N-19004 at low dose reduced cleaved caspase 3-positive cells, although some cell profiles were still detectable in the inner retina. Conversely, retinas from STZ rats treated with the high dose of N-19004 showed no cleaved caspase 3 immunostaining, with an immunoreactive pattern almost comparable to controls (Figure 5C).

**Figure 6:**
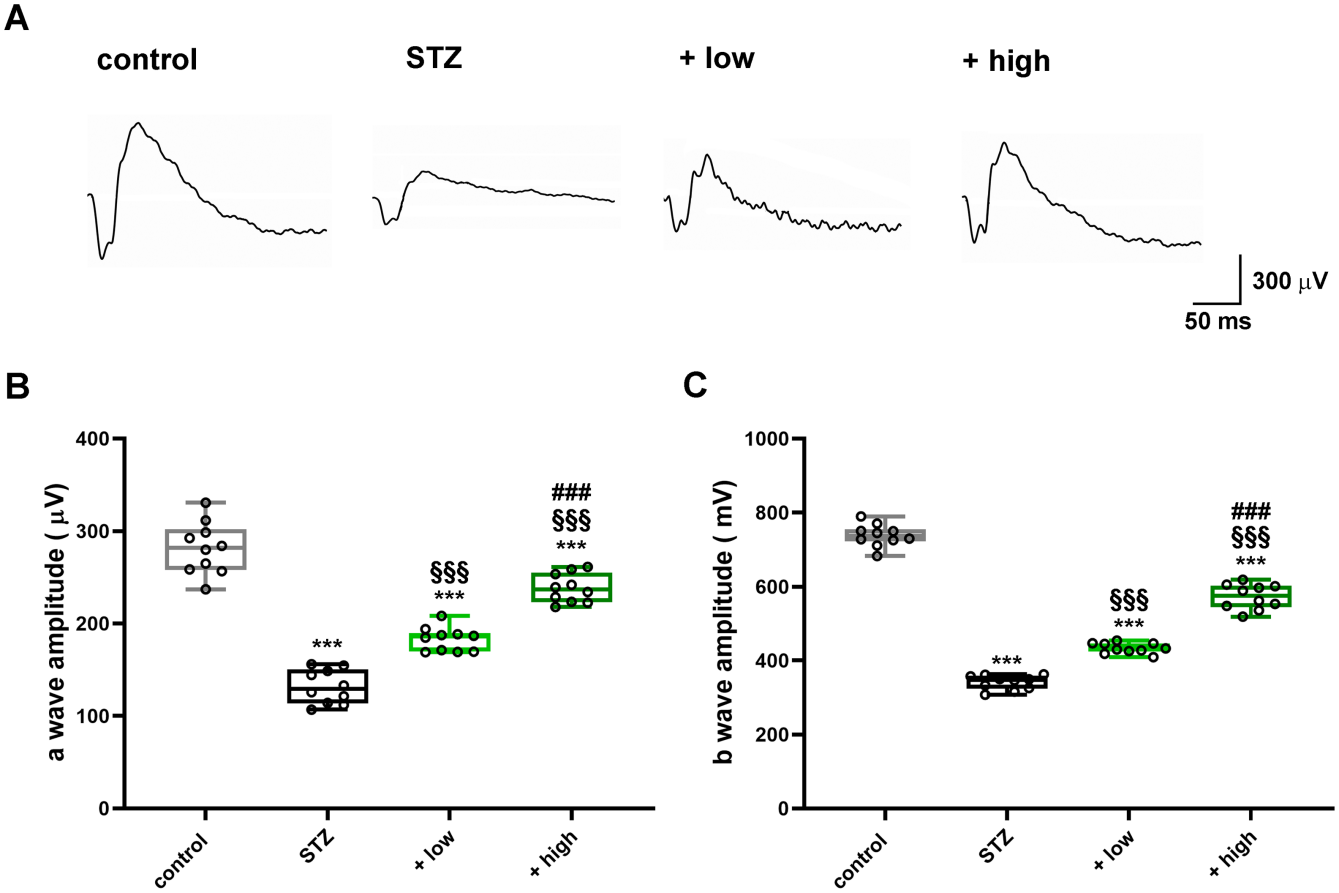
Effects of N-19004 treatment on retinal function. **(A)** Representative scotopic electroretinogram waveforms recorded at 10 cd·s/m^2^ light intensity in control and STZ rats either untreated or treated with N-19004 at low or high dose. **(B,C)** Quantitative analyses of a-wave **(B)** and b-wave **(C)** amplitudes. Data are expressed as box plots with minimum to maximum whiskers. Differences between groups were assessed using one-way ANOVA followed by Tukey’s multiple comparisons *post-hoc* test. *n* = 10 animals per group. *** *p* < 0.001 vs. control; ^§§§^ *p* < 0.001 vs. STZ; ^###^ *p* < 0.001 vs. low dose.

The N-19004-promoted reduction in retinal cell death was also accompanied by an improvement in retinal function, as assessed by full-field scotopic ERG. As shown by the representative waveforms in Figure 6A and the quantitative analyses in Figures 6B,C, untreated STZ rats displayed reduced a-wave and b-wave amplitudes when compared to healthy controls. The administration of N-19004 partially inhibited both a-wave and b-wave amplitude loss in STZ rats with a dose-dependent effect.

## 4 Discussion

The present study provides evidence that N-19004, a novel uPAR antagonist dampening uPAR binding to FPR1, improves retinal cell resilience thus resulting in ameliorated retinal function in the STZ rat model of DR, suggesting that uPAR/FPR1 interactions might contribute to the regulation of neurovascular damage during the early stages of the disease. We demonstrated the anti-inflammatory activity of N-19004, indicated by the decreased production of inflammatory cytokines, thereby alleviating early BRB impairment and improving retinal viability and function under hyperglycemic conditions. Notably, N-19004 appears to exert its beneficial effects independently of glycemic control, which aligns with the action of other uPA/uPAR inhibitors in this model (Cammalleri et al., 2017).

The upregulation of uPAR and its lateral partners, including FPRs, under hyperglycemic conditions has raised the question of whether uPAR/FPRs functional crosstalk may regulate DR progression from its early stages (Navaratna et al., 2008; El-Remessy et al., 2013; Cammalleri et al., 2017; Cammalleri et al., 2017). In particular, the interaction between uPAR and FPRs would result in increased activation of intracellular signaling cascades promoting the expression of molecular mediators including pro-inflammatory cytokines and chemokines, which in turn promote inflammation and compromise BRB integrity under hyperglycemic conditions. In this respect, our previous studies using uPAR-derived tetrapeptide antagonists unraveled that the disruption of uPAR/FPRs interactions in animal models of DR restores BRB integrity and results in improved retinal function possibly through an anti-inflammatory mechanism, thus supporting a pathogenic role of this system in the early establishment of DR-related inflammation (Cammalleri et al., 2017; Cammalleri et al., 2017). Building on this evidence, we recently designed a novel FPR1 antagonist, called N-19004, to study the specific role of uPAR/FPR1 interactions as regulators of inflammatory mechanisms in retinal degenerative disorders (De Fenza et al., 2024; De Fenza et al., 2025). This new compound displays a strong binding affinity for FPR1, as indicated by molecular docking studies demonstrating the deep interaction of its pharmacophores with the receptor binding cavity (De Fenza et al., 2024). Thus, through its intimate binding to FPR1, N-19004 would have the potential to reduce uPAR-FPR1 interactions, leading to the inhibition of FPR1 downstream effectors, including pathways involved in the activation of inflammatory processes.

Given the potential of N-19004 to inhibit the uPAR/FPR1 axis, we tested its efficacy in the STZ rat model, a well-established model that reproduces early pathogenic features of DR, to dissect the role of uPAR/FPR1 interactions in the progression of the disease. In this model, the STZ-mediated loss of pancreatic beta cells triggers oxidative stress and inflammation (Moldovan et al., 2025), generating a pro-inflammatory milieu that promotes glial activation and sustains a chronic inflammatory state (Vecino et al., 2016). This cascade ultimately leads to BRB breakdown and vessel leakage, thereby exacerbating retinal damage (Antonetti et al., 1999). Consistent with previous studies, the amplification of retinal damage induced by BRB breakdown in this experimental model was associated with increased apoptosis, predominantly in the inner retina, that was accompanied by altered retinal function (Li et al., 2008; Sasaki et al., 2010; Thounaojam et al., 2017; Amato et al., 2018; Canovai et al., 2022). As shown by the present findings, the impairment of b-wave amplitude well correlates with the increased apoptotic activity in the inner retina and with Müller cell reactivity observed in STZ rats, suggesting a direct correlation between the presence of apoptotic cells in the inner retinal layers and the reduced function of post-receptoral retinal cells. On the other hand, the reduced a-wave amplitude measured in the absence of overt outer retinal apoptosis may reflect early photoreceptor distress, in line with previous reports demonstrating early degenerative alterations of photoreceptors and the pigment epithelium occurring before the onset of cell death mechanisms (Énzsöly et al., 2014; Liu et al., 2016).

As demonstrated by the present findings, the inhibition of uPAR/FPR1 interaction by N-19004 was sufficient to suppress the activation of inflammatory pathways in the STZ rats. In support of a putative anti-inflammatory action of N-19004, recent studies have reported a strong inhibitory effect of N-19004 on FPR1-mediated cell migration *in vitro*, whereas its systemic administration in a mouse model of choroidal neovascularization restrains the expression of inflammatory molecules, thereby inhibiting the formation of pathological lesions (De Fenza et al., 2024). In addition, N-19004 has shown protective effects in the rd10 mouse model of retinitis pigmentosa, where this molecule significantly prevents photoreceptor degeneration by inhibiting FPR1-related signaling and inflammatory processes (Amato et al., 2025). The anti-inflammatory activity of N-19004 can be also supported by the reduced reactivity of GFAP-positive Müller cell glia observed here, as this cell type contributes to the chronic pro-inflammatory drive in the diabetic retina.

As also shown by the present findings, the anti-inflammatory effect of N-19004 in the diabetic retina is paralleled by the restoration of BRB integrity, the inhibition of apoptotic processes, and a partial recovery of retinal function. Whether the protective effects of N-19004 are due to its action on either vascular or neuronal component remains unclear. On one hand, the effects of N-19004 on ameliorating retinal function may occur as a consequence of the reduction in vascular damage and BRB disruption, as uPAR/FPRs upregulation is known to affect BRB integrity in DR (Cammalleri et al., 2017). In this line, the effect of N-19004 on the vascular component, in particular on BRB dysfunction, would be supported by previous studies showing that inhibiting uPAR/FPRs crosstalk restores tight junctions and reduces vessel leakage in models of oxygen-induced retinopathy and AMD, and dampens VEGF-promoted permeability in human retinal endothelial cells (Motta et al., 2016; Dal Monte et al., 2015; Cammalleri et al., 2016). However, direct evidence of FPR1 expression specifically in retinal endothelial cells is still limited, opening the possibility that vascular protection may also result from indirect modulation of inflammatory or glial responses within the neurovascular unit. On the other hand, an additional direct effect of N-19004 on retinal neuron viability and function cannot be excluded, as FPR1 expression has also been reported in the neuroretina, in particular in the GCL and photoreceptors (Gardner et al., 2017). Overall, these observations would suggest that N-19004 may exert its effects through coordinated modulation of multiple cellular components of the neurovascular unit rather than a single target. Therefore, additional studies using cell-specific approaches will be required to fully dissect the relative contribution of vascular and neuronal effects following the inhibition of uPAR/FPR1 interactions with N-19004.

The anti-inflammatory activity of N-19004 is not correlated with the downregulation of uPAR and FPRs, in line with other inhibitors of uPAR/FPRs interactions in this experimental model (Cammalleri et al., 2017). This would suggest that the mechanism of action of N-19004 would rely on a functional interference with uPAR/FPR1 binding rather than receptor suppression. Therefore, N-19004 action would switch off the intracellular signaling pathways leading to the inactivation of downstream effectors, including transcription factors such as NF-kB and STAT3, that regulate the expression of pro-inflammatory molecules. In this respect, the observed reduction of NF-kB and STAT3 phosphorylation, concomitantly with the downregulation of their pro-inflammatory target genes, supports effective silencing of uPAR/FPR1 downstream signaling despite stable uPAR/FPRs levels. It is clear that more robust evidence of NF-kB activation (and of the effects of N-19004 on this process, as well) may be the evaluation of NF-kB nuclear translocation using a nuclear fractionation assay instead of NF-kB phosphorylation in retinal extracts. However, the parallelism between NF-kB phosphorylation and the levels of NF-kB targets demonstrated here, strongly indicates that N-19004 exerts its effects by reducing NF-kB activation.

Whether the protective effects of N-19004 in the STZ rat model could be exclusively ascribed to its anti-inflammatory properties is not obvious. In effect, FPR1 is a G protein-coupled receptor known to regulate a broad spectrum of downstream signaling pathways potentially involved in a wide array of cell functions. This opens the possibility for additional mechanisms beyond inflammation that may be involved in the therapeutic effects of N-19004 and whose contribution would require further investigations. In this regard, we have previously demonstrated that N-19004 treatment also exerts antioxidant activities in rd10 mice, possibly by potentiating the endogenous antioxidant response to counteract oxidative stress (Amato et al., 2025). Given that oxidative stress is a central pathogenic trigger in DR (González et al., 2023), the possibility exists that the antioxidant properties of N-19004 may also contribute to its protective efficacy in the STZ model.

Although the available therapies to counteract DR are mainly based on intravitreal injections, we chose to administer N-19004 using a systemic route since, in a translational perspective, the possibility of using N-19004 through this route may ensure better patient compliance than intravitreal injections. Using the body surface area normalization method for an allometric dose translation (Reagan-Shaw et al., 2008) we can hypothesize that the higher systemic dose used in rats (12 mg/kg) might be scaled down to about 1.9 mg/kg for humans. On the other hand, with the aim of maximizing ocular bioavailability and minimize systemic exposure, this dose might be further reduced using local delivery through intravitreal injections. Although the effects of N-19004 in retinopathy models have never been tested by intravitreal injections, we are confident that this route may be as effective as the systemic one in counteracting retinal degeneration in the STZ model. This assumption comes from our previous experience with UPARANT, the uPAR/FPR antagonist from which N-19004 was derived. Intravitreal UPARANT was indeed effective in counteracting angiogenesis in mouse models of oxygen-induced retinopathy, choroidal neovascularization, and rubeosis iridis (Dal Monte et al., 2015; Cammalleri et al., 2016; Locri et al., 2019; Locri et al., 2020). In particular, in a mouse model of rubeosis iridis, UPARANT mitigated angiogenesis in a similar fashion when administered either subcutaneously or intravitreally (Locri et al., 2019).

## 5 Conclusion

At present, the options available for the treatment of DR that are currently in use are administered at later stages of the disease, when retinal dysfunction has become severe and difficult to treat. Our findings indicate that the inhibition of uPAR/FPR1 interactions with the novel synthetized compound N-19004 reduces the early BRB breakdown and promotes retinal cell resilience, thus mitigating retinal dysfunction in the STZ rat model of DR. The protective efficacy of N-19004 in this model may be related, at least in part, to its anti-inflammatory activity, although other cellular mechanisms affected by N-19004 need to be investigated in further studies. Although limited to pre-clinical context, the anti-inflammatory activity of N-19004 might open the possibility for the development of strategies targeting the uPAR/FPR1 system for the management of DR at its early stages, in order to delay the progression of the disease. The extrapolation of these results into the clinic is not straightforward, as the STZ model may not fully recapitulate all the pathological signs in the human DR, including macular edema and proliferative events, thus rendering this model suitable for the early phases of the disease which are, however, often asymptomatic in humans.

## Data Availability

The original contributions presented in the study are included in the article/supplementary material, further inquiries can be directed to the corresponding author.
